# The vaccination rift effect provides evidence that source vaccination status determines the rejection of calls to get vaccinated

**DOI:** 10.1038/s41598-022-23291-w

**Published:** 2022-11-08

**Authors:** J. Lukas Thürmer, Sean M. McCrea

**Affiliations:** 1 Political and Intercultural Psychology Group, Department of Psychology, Paris-Lodron University Salzburg, Hellbrunnerstr. 34, 5020 Salzburg, Austria; 2 Department of Psychology, University of Wyoming, Laramie, USA

**Keywords:** Psychology, Human behaviour, Epidemiology

## Abstract

COVID vaccination protects individuals and helps end the pandemic, but a sizable minority in Western countries rejects the vaccine. Vaccination status should serve as a group membership, critical communication between groups undermines trust, and we accordingly suggest that calls to get vaccinated by vaccinated sources lead to defensive rejection instead of desired behavior change. We term this the *vaccination rift effect*. A unique collaboration with national print, online and TV news media yielded a large (*N* = 1170), age-representative sample of Austrian citizens for our fully randomized experiment. Participants exhibited the vaccination rift: They ascribed less constructive motives, *d* = 0.28, 95% CI [0.17; 0.40], experienced more threat, *d* = − 0.30, 95% CI [− 0.42; − 0.19], and ascribed worse personality characteristics to vaccinated (vs. unvaccinated) commenters, *d* = 0.17, 95% CI [0.06; 0.29]. Constructiveness consistently predicted behavioral measures of counterarguing and vaccination planning (indirect effects *B* = 0.033, *SE* = 0.013 and *B* = − 0.056, *SE* = 0.014). The vaccination rift was substantially stronger among the critical group of unvaccinated participants, *d*s = |0.39–0.52|, than among those fully vaccinated, *d*s = |0.08–0.17|. We discuss how to apply these psychological mechanics of the vaccination rift to public campaigns.

## Introduction

Dr. Francis Collins, outgoing director of the National Institutes of Health noted that he “never imagined a year ago, when those vaccines were just proving to be fantastically safe and effective, that we would still have 60 million people who had not taken advantage of them because of misinformation and disinformation,” concluding that NIH underinvested in understanding human behavior^1^. Despite vast evidence that COVID-19 vaccines are safe and effective^2^, a substantial minority of the population in Western countries foregoes available opportunities to get vaccinated. Such vaccine hesitancy^3–5^ to a large degree depends on “inadequate or poor immunization program communications” (p. 4163)^6^.

We take a new group-level perspective to identify vaccination status of the communicator as a key impediment for common immunization program communications. Specifically, vaccination status may serve as an indicator of group membership, undermining trust in the content of vaccinated commenter’s message due to suspicions of their motive. We term this rejection of calls to get vaccinated voiced by vaccinated sources the *vaccination rift effect* and test it in a high-powered field experiment in a population largely unaffected by structural barriers to vaccination (Austria).

### Vaccine hesitancy and Covid-19

Before any Covid-19 vaccines were available, medical researchers urgently called for “[i]nterventional educational campaigns targeted towards populations at risk of vaccine hesitancy.” (p. 1)^7^, due to fears that vaccine uptake would otherwise be insufficient. Unfortunately, these reservations proved true. Western countries with sufficient public access to vaccines have insufficient vaccination rates^3^. A recent review of the psychological underpinnings of COVID-19 vaccine uptake has identified one key factor that promotes the decision to get vaccinated: Trust in the health authorities that recommend getting vaccinated^8–10^. This conclusion is also in line with past research on vaccine hesitancy that has identified *confidence* in vaccines and the system that delivers them is one of the keys to vaccine uptake^11^. Accordingly, messages about Covid-19 vaccines need to build trust. Common vaccine communication strategies seek to address this issue at the interpersonal, individual, and organizational levels^3^, for instance by using expert communicators.

These existing accounts overlook the fact that vaccination has become a matter of established group memberships. For instance, correlational research indicates that Republicans in the United States were significantly more likely to reject COVID-related health measures^12^, even leading to more observed deaths due to COVID in Republican-leaning areas^13^. Facets of conservativism were also related to vaccine hesitancy in multiple European countries^14^. Moreover, the homogenous nature of social networks may further radicalize attitudes about vaccination^15^. These correlational studies were unable to clarify the causal direction of these effects. But they consistently indicate that the decision to get vaccinated is socially determined^4^, that is, by group membership.

We challenge the assumption of past research that vaccine hesitancy is characteristic of certain groups and uncover a basic mechanism underlying the social determinants of vaccine hesitancy: The vaccination rift effect. We argue that heated debates around whether to vaccinate may create a social boundary between each side. If vaccination status itself serves as a signifier of social group membership, rifts in communication across these lines will result. Specifically, there is a lack of trust in critical communication that originates from groups other than the one criticized^16–18^. The rejection of intergroup criticism should undermine calls to get vaccinated by vaccinated sources.

In line with our view, the general perception that society is divided leads to lack of trust^19^, especially when members of different (sub-)groups convey critical messages. Although critical comments from fellow group members are tolerated, criticism that is voiced by members of an outgroup are consistently rejected by group members and bystanders^18^. Perceivers will even invest their own money to punish intergroup criticism^16,17^. Intergroup criticism violates established conversational norms, and so perceivers do not trust the motives of outgroup critics^16,20^. Importantly, according to recent research, even members of uninvolved groups or members of the commenter’s group are suspicious of intergroup criticism^16,17,21^. That is, the rejection of intergroup criticism extends to comments directed at groups one does not belong to. These findings strongly suggest that intergroup communication advocating for vaccination may undermine trust in the vaccine, potentially even among those using it (bystanders). That is, calls to get vaccinated by a vaccinated person should lead to skepticism and rejection of the message rather than promoting the desired behavior (i.e., a vaccination rift). Conversely, calls to get vaccinated by an unvaccinated person may be more effective because within-group criticism does not violate conversational norms.

Vaccine hesitancy accounts have been criticized for drawing attention away from structural barriers to vaccination^4^, such as poor public health funding, insufficient communication or incentives, or low vaccine availability. To account for these concerns, we conducted our study in a population that likely does not suffer from these barriers to vaccination. Austria consistently ranks among the top 15% of countries in the world in terms of per capita GDP (top 11% 2020), income equality (Gini index of family income distribution, top 14%), and has universal healthcare which leads to very good population health (e.g., 5 maternal and 3.3 child deaths per 100,000 births, top 11% and top 10%, respectively)^22^. During the COVID 19 pandemic, Austria provided its citizens with free testing and vaccination at multiple sites, and with multiple additional incentives to get vaccinated (e.g., a raffle for those who got vaccinated and drop-in vaccinations at accessible and attractive locations). In fact, the government had announced a vaccine mandate on Dec 6, 2021 to be effective Feb 1, 2022, further providing incentives to get vaccinated at the time of the study. These low barriers to vaccination in Austria rule out access barriers^4^ as an alternative explanation for the expected vaccination rift effect.

In the present research, we focus on the vaccination rift effect in calls to get vaccinated between people with different vaccination status. In a unique cooperation with national newspaper and television, we recruited a large sample of Austrians (*N* = 1170) that was age-representative. Crucially, we managed to over-sample the hard-to-reach critical group of unvaccinated participants, leading an approximately even number of fully vaccinated and unvaccinated participants. Participants responded to media announcements in a national newspaper (11/29/2021), on television (12/26/2021), or respective online articles. In a fully randomized online experiment, participants read a critical comment calling to get vaccinated against COVID. We attributed this critical comment either to an unvaccinated person pledging to get vaccinated soon (unvaccinated source condition) or an already fully vaccinated person (vaccinated source condition). Participants then indicated the motive of the commenter regarding the people in Austria, how threatening the message was, and their evaluations of the commenter. To assess the behavioral impact of these messages, we assessed participants’ vaccination planning behavior (i.e., requesting additional information on where to get vaccinated) and counterarguing (i.e., free-text response on their opinion of the vaccine). Planning and behavioral engagement are proximal predictors of health behavior, including responses during COVID^23^, and thus good behavioral indicators of the proposed vaccination rift effect. Our data are available at https://osf.io/c8tn3/. We did not preregister this experiment due to time constraints.

## Results

Our sample (*M* = 49.09, *SD* = 13.32, range [15; 83]) was representative of the adult population age average (*M* = 49.74), *t*(1169) = − 1.68*, p* = 0.094,* d* = − 0.05. However, we oversampled female respondents (57% vs. 52% in the population, *z* = 3.43, *p* < 0.001) and undersampled male respondents (43% vs. 48% in the population, *z* = − 3.43, *p* < 0.001). Females commonly report more positive vaccine attitudes and have higher vaccine uptake. Consequently, females should be less likely to exhibit the vaccination rift effect and our sample thus provided a critical test of our hypotheses. Fully vaccinated participants were represented well (40% vs. 40% in the population as of 12/26/2021, *z* < 0.01, *p* > 0.999), but we undersampled partly vaccinated or recovered participants (29% vs. 34% in the population, *z* = − 3.63, *p* < 0.001), in exchange for a greater representation of the key group of unvaccinated participants (32% vs. 26% in the population, *z* = 4.65, *p* < 0.001).

### Confirmatory analyses

**Figure 1:**
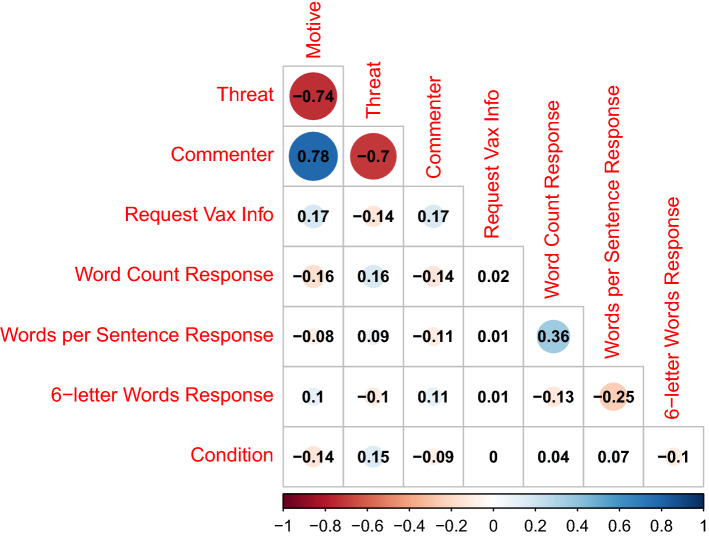
Correlation plot of dependent variables and condition.

We observed overall main effects in line with the proposed vaccination rift effect (correlations in Fig. 1). Participants ascribed less constructive motives, *t*(1167.50) = 4.80, *p* < 0.001, *d* = 0.28, 95%CI [0.17; 0.40], and less positive personality characteristics, *t*(1157.08) = 2.92, *p* = 0.004, *d* = 0.17, 95%CI[0.06; 0.29], to vaccinated commenters than to unvaccinated commenters, despite identical message content. Participants also overall reported feeling more threatened by calls to get vaccinated from the vaccinated source than the unvaccinated source, *t*(1161.92) = − 5.17, *p* < 0.001, *d* = − 0.30, 95%CI[− 0.42; − 0.19] (Table 1).

**Table 1:** Mean comment ratings and by comment source (collapsed across participant vaccination status).

	Unvaccinated source	Vaccinated source	*t*(1169)	*p*	*d*	95% CI
**Comment ratings**						
Message motive rating	4.08 (1.78, [1; 7], − 0.02)	3.57 (1.81, [1; 7], 0.30)	4.80	< 0.001	0.28	[0.17; 0.40]
Message threat rating	3.37 (1.71, [1; 7], 0.28)	3.90 (1.83, [1; 7], − 0.06)	− 5.17	< 0.001	− 0.30	[− 0.42; − 0.19]
Commenter rating	3.12 (1.45, [1; 7], 0.50)	2.86 (1.59, [1; 7], 0.84)	2.92	0.004	0.17	[0.06; 0.29]

Standard deviations, range, and skew are in parentheses. Reporting adjusted estimates for Cohen's *d.*

We analyzed participants behavioral planning and counterarguing in free text as behavioral indicators. Only 15.6% of participants requested additional information and the comment source had no significant direct impact on this measure, χ^2^(1, *N* = 1170) < 0.01, *p* > 0.999. We expected that criticism from the vaccinated source would elicit more counterarguing as evidenced by a greater wordcount of the free-text response (assessed in LIWC2015^24^). Past research has observed that response length is highly correlated with negative content when counterarguing group criticism^25^, and word count thus provides a good behavioral indicator of the vaccination rift. Participants responding to the vaccinated commenter indeed used more words to express their opinion about the vaccine (*Mdn* = 22.50) than did participants responding to the unvaccinated commenter (*Mdn* = 19.00), Wilcoxon signed-rank tests *W* = − 3.00, 95%CI [− 5.00; − 1.00], *p* = 0.009, *r* = 0.08 (Table 2).

**Table 2:** Behavioral measures by comment source (collapsed across participant vaccination status).

	Unvaccinated source	Vaccinated source	*W*	*p*	*r*	Hodges–Lehmann	95% CI
**Behavioral vaccination planning**							
Request further information [Frequency (%)]	79 of 507 (15.58%)	79 of 505 (15.64%)	–	*ns*	–	–	–
**Behavioral counterarguing**							
Length of response (word count) [Median (standard deviation, range, skew)]	19.00 (54.83, [0; 514], 3.69)	22.50 (73.90, [0; 1098], 7.92)	− 3.00	0.009	0.08	− 3.00	[− 5.00; − 1.00]

### Exploratory analyses

#### Participant vaccination status

**Figure 2:**
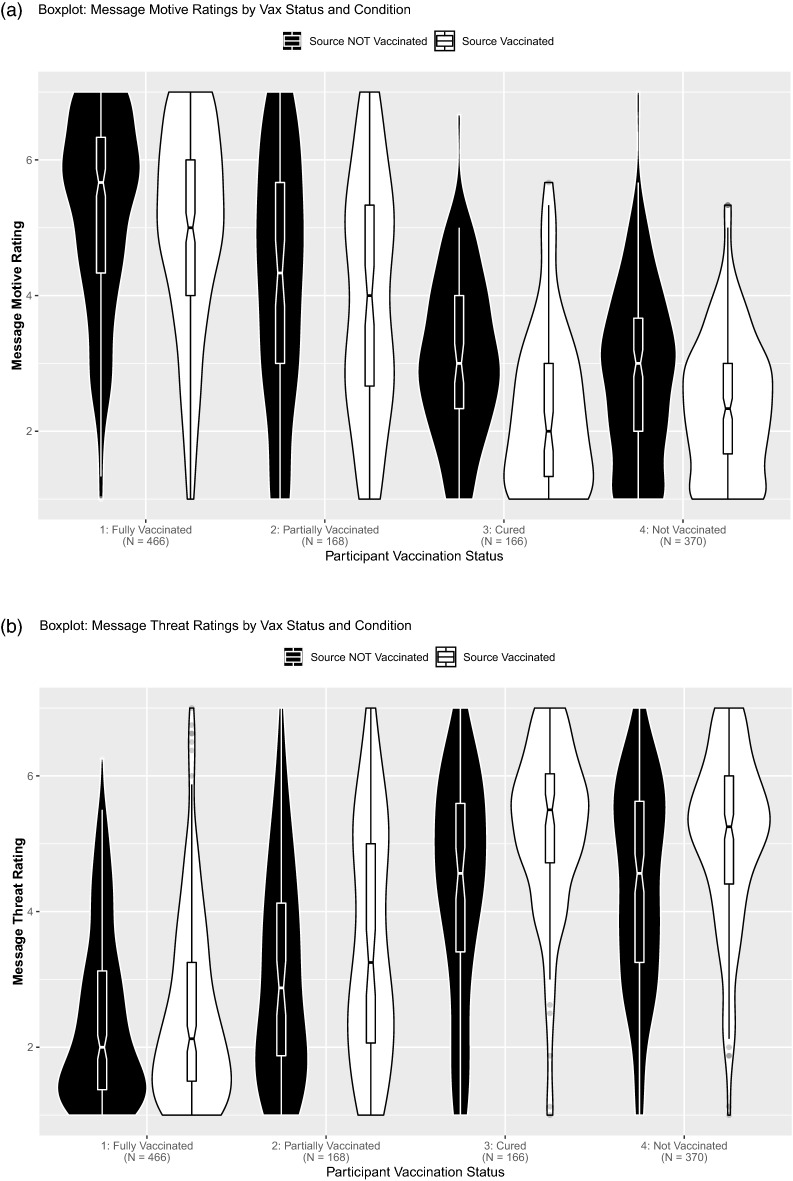

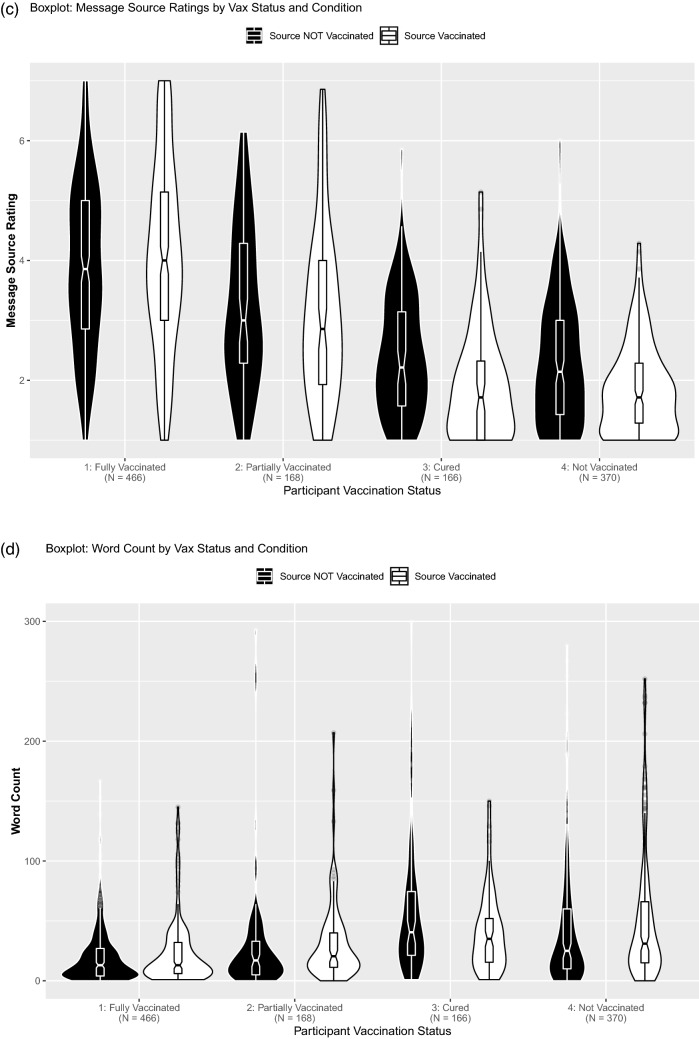
Boxplots with violin-plots (**a**) Message motive rating by message source (unvaccinated vs. vaccinated) and participant vaccination status. (**b**) Message threat by message source (unvaccinated source vs. vaccinated) and participant vaccination status. (**c**) Message source rating by message source (unvaccinated source vs. vaccinated) and participant vaccination status. (**d**) Word count of free text response by message source (unvaccinated source vs. vaccinated) and participant vaccination status. Eight extreme datapoints > 300 words were omitted for plotting. *Note.* Boxplot notches indicate 95% CIs.

Vaccination status may moderate the rift effect because those who have already adopted the behavior are less likely to be suspicious of the message, even though past research indicates that even members of the commenter’s group are suspicious of intergroup criticism^21^. Indeed, participants’ own vaccination status moderated the observed effects on message threat (Fig. 2b) and commenter evaluation (Fig. 2c), as indicated by significant Message Source × Participant Vaccination Status interactions, *F*(2, 1164) = 3.94, *p* = 0.008, η_p_^2^ = 0.01, and *F*(2, 1164) = 5.40, *p* = 0.001, η_p_^2^ = 0.01. This interaction was not significant for comment motive (Fig. 2a), *F*(2, 1164) = 1.46, *p* = 0.225, η_p_^2^ < 0.01. However, across all three measures, pairwise comparisons (all Tukey adjusted) indicated that the vaccination rift was significant for unvaccinated, *p*s = 0.002 to < 0.001, and recovered participants, *p*s = 0.014 to < 0.001 (group-wise tests and effect sizes in Table 3). Those fully vaccinated showed a vaccination rift for motive, *p* = 0.049, but not threat, *p* = 0.444, or commenter ratings,* p* = 0.162. Partly vaccinated participants did not show a vaccination rift for motive, *p* = 0.181, or commenter evaluations, *p* = 0.479, but for threat, *p* = 0.032. In sum, we observed a consistent vaccination rift among those unvaccinated or recovered but not those (partly) vaccinated.

**Table 3:** Mean comment ratings by comment source for each vaccination status participant group.

	Unvaccinated source	Vaccinated source	*t*(1169)	*p*	*d*	95% CI
**Unvaccinated participants (*N* = 370)**						
Message motive rating	2.83 (1.28, [1; 7], 0.35)	2.38 (1.02, [1; 5.333], 0.46)	3.10	< 0.001	0.39	[0.18; 0.60]
Message threat rating	4.41 (1.50, [1; 7], − 0.35)	5.09 (1.28, [1; 7], − 0.80)	− 4.63	< 0.001	− 0.49	[− 0.70; − 0.28]
Commenter rating	2.30 (1.04, [1; 6], 0.79)	1.83 (0.73, [1; 4.286], 0.90)	3.64	< 0.001	0.52	[0.31; 0.73]
**Recovered participants (*N* = 166)**						
Message motive rating	3.06 (1.18, [1; 6.667], 0.23)	2.30 (1.19, [1; 5.667], 0.86)	3.51	< 0.001	0.64	[0.32; 1.06]
Message threat rating	4.38 (1.61, [1; 7], − 0.54)	5.16 (1.29, [1; 7], − 1.01)	− 3.57	< 0.001	− 0.54	[− 0.85; − 0.22]
Commenter rating	2.40 (1.00, [1; 5.857], 0.66)	1.92 (0.93, [1; 5.143], 1.21)	2.46	= 0.014	0.49	[0.18; 0.80]
**Partly vaccinated participants (*N* = 168)**						
Message motive rating	4.25 (1.80, [1; 7], − 0.26)	3.96 (1.73, [1; 7], 0.00)	1.34	= 0.181	0.16	[− 0.14; 0.47]
Message threat rating	3.06 (1.56, [1; 7], 0.52)	3.53 (1.71, [1; 7], 0.23)	− 2.15	= 0.032	− 0.29	[− 0.59; 0.02]
Commenter rating	3.21 (1.35, [1; 6.143], 0.33)	3.07 (1.44, [1; 6.857], 0.67)	0.71	= 0.479	0.10	[− 0.21; 0.40]
**Fully vaccinated participants (*N* = 466)**						
Message motive rating	5.23 (1.44, [1; 7], − 0.78)	4.98 (1.51, [1; 7], − 0.63)	1.98	0.049	0.17	[− 0.01; 0.36]
Message threat rating	2.40 (1.27, [1; 6.25], 0.90)	2.50 (1.37, [1; 7], 1.13)	− 0.77	0.444	− 0.08	[− 0.26; 0.11]
Commenter rating	3.90 (1.41, [1; 7], 0.07)	4.06 (1.55, [1; 7], 0.09)	− 1.40	0.162	− 0.11	[− 0.29; 0.07]

Standard deviations, ranges, and skew are in parentheses. Reporting adjusted estimates for Cohen's *d.*

Effects on requesting additional information or written responses (Fig. 2d) were not moderated by participants’ vaccination status, *p*s > 0.454 (group-wise tests and effect sizes in Tables 4 and 5). However, ironically, those participants who were already fully vaccinated (19%) were significantly more likely to request additional information than those unvaccinated (08%), χ^2^(1, *N* = 836) = 20.66, *p* < 0.001, or recovered (07%), χ^2^(1, *N* = 632) = 11.98, *p* = 0.001. Partly vaccinated participants were also significantly more likely to request additional information than those unvaccinated (08%), χ^2^(1, *N* = 538) = 8.60, *p* = 0.003, or recovered (07%), χ^2^(1, *N* = 334) = 6.19, *p* = 0.013. No significant difference between partly and fully vaccinated participants, χ^2^(1, *N* = 634) = 0.34, *p* = 0.562, or recovered and unvaccinated participants emerged, χ^2^(1, *N* = 536) < 0.01, *p* = 0.945. Fully vaccinated participants provided shorter responses than recovered, *p* < 0.001, or unvaccinated participants, *p* < 0.001. Likewise, partly vaccinated participants provided shorter responses than recovered, *p* = 0.018, or unvaccinated participants, *p* = 0.034. Length of response neither differed between fully and partly vaccinated participants, *p* = 0.200, nor recovered and unvaccinated participants, *p* = 0.888. In all, the critical group of the unvaccinated exhibited the strongest vaccination rift effect (detailed results in Appendix 1).

**Table 4:** Behavioral planning by comment source for each vaccination status participant group.

	Unvaccinated source	Vaccinated source	χ^2^(1)	*p*
**Unvaccinated participants (*N* = 370)**				
Request further information	11 of 176 6.25%	18 of 184 9.78%	0.79	0.374
**Recovered participants (*N* = 166)**				
Request further information	6 of 82 7.32%	6 of 84 7.14%	< 0.01	> 0.999
**Partly vaccinated participants (*N* = 168)**				
Request further information	15 of 77 19.48%	13 of 91 14.29%	0.48	0.489
**Fully vaccinated participants (*N* = 466)**				
Request further information	47 of 251 18.73%	42 of 215 19.53%	0.01	0.918

Standard deviations, ranges, and skew are in parentheses. Reporting adjusted estimates for Cohen's *d.*

**Table 5:** Counterarguing by comment source for each vaccination status participant group.

	Unvaccinated source	Vaccinated source	*W*	*p*	*r*	Hodges-Lehmann	95% CI
**Unvaccinated participants (N = 370)**							
Length of response (word count)	51.44 (74.30, [0; 514], 3.17)	53.42 (58.01, [0; 354], 1.99)	15,068	0.051	0.10	− 5.00	[− 12.00; 0.00]
**Recovered participants (*N* = 166)**							
Length of response (word count)	59.82 (57.44, [1; 300], 1.77)	53.67 (120.02, [1; 1098], 7.85)	3964	0.093	0.13	8.00	[− 1.00; 19.00]
**Partly vaccinated participants (*N* = 168)**							
Length of response (word count)	31.78 (53.16, [0; 293], 3.49)	40.77 (86.03, [0; 786], 7.25)	2865.5	0.042	0.16	− 5.00	[− 10.99; 0.00]
**Fully vaccinated participants (*N* = 466)**							
Length of response (word count)	21.63 (26.07, [0; 167], 2.52)	28.23 (52.73, [1; 549], 6.63)	25,366	0.265	0.05	− 1.00	[− 3.99; 0.99]

Standard deviations, ranges, and skew are in parentheses. Reporting adjusted estimates for Cohen's *d.*

#### Structural equation modelling

**Figure 3:**
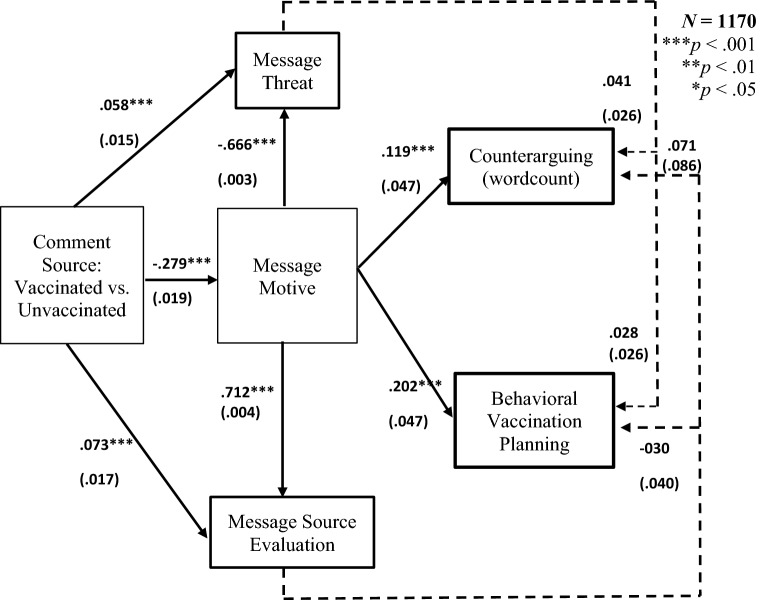
Results of structural equation model analyses. Dashed lines are non-significant paths. Estimates are standardized regression coefficients and SEs in parentheses.

**Figure 4:**
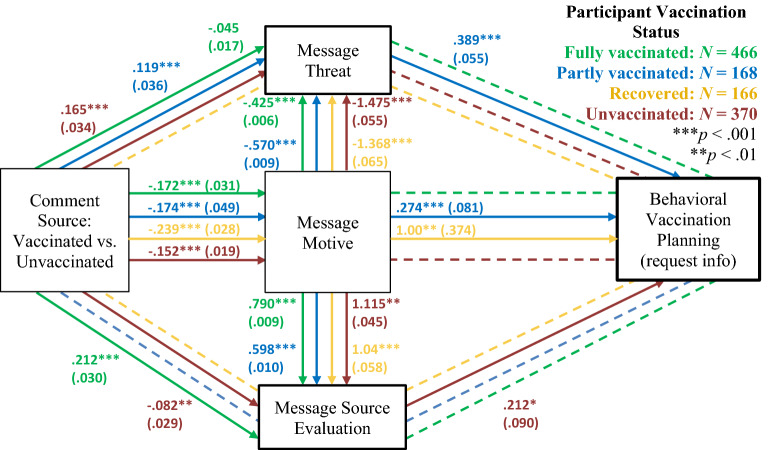
Results of structural equation model analyses on behavioral planning by vaccination status. Dashed lines represent non-significant paths. Estimates are standardized regression coefficients and SEs in parentheses; only significant paths are reported.

**Figure 5:**
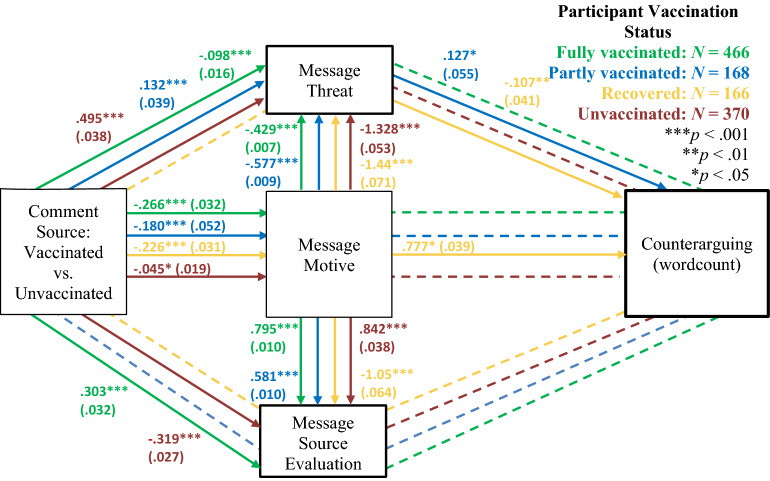
Results of structural equation model analyses on counterarguing by vaccination status. Dashed lines represent non-significant paths. Estimates are standardized regression coefficients and SEs in parentheses; only significant paths are reported.

We next explored potential mechanisms underlying the vaccination rift effect (Fig. 3; see Appendix 1, for detailed results). Comment source predicted attributed comment constructiveness, which predicted higher odds that participants engaged in behavioral planning as well as *more* counterarguing. The indirect effects from message source to planning behavior and counterarguing via motive were significant. Subsamples analyses (Figs. 4 and 5) showed that none of the mediators predicted planning or counterarguing among fully vaccinated participants. Among partly vaccinated participants, a serial indirect effect as well as simple serial effects via message constructiveness and threat emerged on planning but not counterarguing. Among recovered participants, an indirect effect on behavioral planning via message motive emerged as well as on counterarguing via motive and threat. Finally, among unvaccinated participants, a serial indirect effect from message constructiveness via commenter evaluations on behavioral planning (but not counterarguing) emerged. Apparently, for the unvaccinated, trust in the source is particularly important determinant for vaccination rift effects on behavioral planning.

## Discussion

Epidemiological research indicates that vaccine hesitancy is a key factor in prolonging the current COVID pandemic^2^. Here, we propose a group-level perspective on vaccine hesitancy and observed that vaccination status divided participants into “them” and “us”, substantially reducing trust in calls to get vaccinated. This vaccination rift effect was strongest among those unvaccinated, and also emerged on a behavioral measure of counterarguing. Few people (and ironically, especially few of those not fully vaccinated) engaged in behavioral planning and no direct vaccination rift effect emerged on this measure. Indirect effects on behavioral planning emerged via message motive, although follow-up analyses indicate that these mediation patterns differed according to participant vaccination status.

We recruited our large sample from the general population in Austria, a country with very low structural barriers to vaccination. While ruling out structural factors as an alternative explanation, one may question the generalizability of our findings beyond this specific population. However, the rejection of outgroup criticism has been observed consistently in other countries, including the US, Australia, and Germany^20,26^. We also did not assess whether participants actually got vaccinated in response to the calls. However, we did include a measure of behavioral planning, which is a proximate predictor of actual behavior^27^. Finally, we did not pre-register our study. However, two recent registered reports observed the consistent rejection of outgroup criticism^16,17^, attesting to the robustness of the hypothesized effects. We nevertheless encourage independent replications of the vaccination rift effect in different populations and directly assessing vaccine uptake.

Our findings apply to ongoing messaging campaigns. Campaigns commonly use expert sources, such as doctors or medical experts, which has been shown to increase message effectiveness^28^. Our observed effects did not hinge on source characteristics other than vaccination status, and thus extend known effects of message effectiveness. What is more, the common practice of highlighting that messages sources have been vaccinated may even backfire by eliciting considerable defensiveness rather than goal-directed behavior. This interpretation comports well with vaccine hesitancy literature that finds that educational tools all too often have no effect or may even increase vaccine hesitance^29^. Applied in these settings, the small-to-medium participant-level effects observed in the current experiment may elicit substantial population-level increments of vaccine message acceptance.

A second implication of the current research is that trust is key for crafting convincing vaccination messages^8,10^. However, our exploratory analyses indicate that different aspects of trust may be important for different demographics. While perceived comment constructiveness was key to seeking further information overall, recovered as well as partly vaccinated participants’ threat perceptions and unvaccinated participants’ commenter evaluations were better predictors of behavior in these subgroups. Our research suggests that relatively minor changes of calls to get vaccinated may increase their effectiveness. These changes in vaccine messaging are independent of commonly applied strategies and can thus easily be implemented in ongoing targeted campaigns at scale.

### Conclusion

Our research shows that communication about health interventions, such as the COVID vaccine, needs to take group boundaries into account. We are clearly all in this together. But we first need to overcome the vaccination rift, that is, have constructive debates and find inclusive messages. Because we clearly cannot master the current challenges as “them” versus “us”.

## Method

### Participants and design

We collected data between 11/29/2021 and 01/12/2022. Although formal sample size planning was not possible in the current setting, we sought to recruit at least 1000 participants. To advertise the study, the national newspaper “Salzburger Nachrichten” published a brief article on 11/29/2021 (online and print) and the public television station “ORF Salzburg” aired a brief item during the evening news “Salzburg heute” and published an article on their website on 12/26/2021. Both adverts highlighted that we sought to understand how people form their opinion about the Covid-19 vaccine (“Was denkt Österreich?”). All participants provided informed consent (see below) and all ethical standards as put forth by the Declaration of Helsinki and the American Psychological Association were followed. The University of Salzburg internal review board approved this study (IRB number GZ 10/2020).

A total of 1223 participants completed our study (our dataset contains 1599 incomplete study entries; one participant withdrew consent after the study; their responses were located based on given responses and removed from the dataset before commencing with analyses). Forty-seven participants were excluded for failing one or more attention checks (see below) and 9 reported not currently living in Austria (3 of whom also failed manipulation checks), leaving *N* = 1170 for analysis (501 male, 663 female, 4 non-binary, 4 divers; *M*_age_ = 49.09, *SD* = 13.32, range [15; 83]; 1115 Austrian nationality; unvaccinated *N* = 370; recovered *N* = 166; single-dose vaccinated *N* = 15; double-dose vaccinated *N* = 153; fully vaccinated/triple-dose *N* = 466). Participants were randomly assigned to an unvaccinated source or a vaccinated source condition by the survey software formR^30^.

### Materials and procedure

Participants responded to the advertisements “Was denkt Österreich?” (“What does Austria think?”) by clicking on a web-link or using a QR-code that led to a web-based survey in formR^30^. Participants read that we need their help in rating comments by previous participants in a study on people’s thoughts about the COVID vaccine and learn about their personal opinion. Participants read critical comments calling for unvaccinated people to get the COVID vaccine (Appendix 2). To manipulate the message source, the comment was attributed to a former participant who was described as either being unvaccinated but intending to get vaccinated (unvaccinated source condition) or being fully vaccinated (vaccinated source condition). After reading each comment, participants indicated the vaccination status of the commenter and the target group of the comment, as manipulation checks, and responded to the comment. Specifically, participants rated message motive towards Austrians (3 items: To what extent do you think…the comments were constructive; …the person who wrote these comments cares about the people in Austria; …the comments were made in Austrian’s best interest?), the message threat (8 items: To what extent do you think this comment is: threatening, disappointing, irritating, offensive, insulting, hypocritical, judgmental, arrogant), and their evaluation of the commenter (7 items: To what extent did you find the person who wrote the comment: intelligent, trustworthy, friendly, open-minded, likable, respected, interesting). All items were answered on seven-point scales (1: *not at all* to 7: *very much*) and adapted from Hornsey and Imani^20^. After these ratings, participants indicated if they wanted to receive more information on where to get vaccinated (yes/no) and responded in free-text to the question: “What is your personal opinion about the Corona vaccination?” The request of further information and the content of their free-text responses served as behavioral dependent measure.

Finally, participants provided their nationality and Austrian county of residence, their vaccination status (3 doses, 2 doses, 1 dose, unvaccinated, recovered), their age, gender, and nationality, and were debriefed. In accordance with the recommendations of the ethics committee, all participants were provided with official information on the COVID vaccine as well as the link to the official website oesterreich-impft.at, which provides information about the vaccine and vaccination sites.

### Analyses

We used R^31^ in R-Studio^32^ including the packages psych^33^, schoRsch^34^, lmerTest^35^, and lavaan^36^ to analyze our data. We computed single indexes for message motive (α = 0.88), message threat (α = 0.92), and commenter evaluations (α = 0.94).

To investigate the role of participants’ own vaccination status, we clustered them into four groups: Fully vaccinated (3 doses; *n* = 466), partly vaccinated (1 dose, 2 doses; *n* = 166), recovered (*n* = 168), and unvaccinated (*n* = 370). The booster campaign was ongoing during the time of data collection and those participants strongly supporting the vaccination likely already had received the third injection. To test the effect of the vaccination group on the ISE, we computed generalized linear models using lmerTest^35^ including comment source and participants’ vaccination status, as well as the interaction term as fixed effects and each dependent measure in turn (message motive, message threat, commenter evaluation). For behavioral planning, we performed parallel analyses using binomial modeling. To test potential indirect effects of commenter vaccination status on participants’ responses, we conducted a structural equation model analysis using the lavaan package^36^ to test our mediational model (Fig. 2).

## Supplementary Information

Supplementary Information.

## Data Availability

Our data, materials, and analyses are available at https://osf.io/c8tn3/.
