# Supplementary material for: The vaccination rift effect provides evidence that source vaccination status determines the rejection of calls to get vaccinated
Source: Sci Rep. 2022 Nov 8;12:18947. doi: 10.1038/s41598-022-23291-w (PMC9643387; doi:10.1038/s41598-022-23291-w)
Supplement: Supplementary file 1 — Supplementary Information. [file 41598_2022_23291_MOESM1_ESM.pdf]

Appendix 1: Detailed Results of Exploratory Analyses

Participant Vaccination Status

We explored the moderating role of participants' own vaccination status by computing 2 Message Source (vaccinated vs. unvaccinated) × 2 Participant Vaccination Status (fully vaccinated vs. partly vaccinated vs. recovered vs. unvaccinated) ANOVAs using type II sum of squares. Including message motive in the ANOVA yielded main effects of source, $F(1, 1162) = 23.26$, $p < .001$, $\eta_p^2 = .02$, and participants' vaccination status, $F(3, 1162) = 265.91$, $p < .001$, $\eta_p^2 = .41$. No Message Source × Participant Vaccination Status interaction emerged, $F(3, 1162) = 1.46$, $p = 225$, $\eta_p^2 = < .01$. Pairwise comparisons of the participants' vaccination status showed that vaccinated participants evaluated the comment as more constructive than partly vaccinated participants, $p < .001$, and partly vaccinated participants evaluated the comment as more constructive than unvaccinated participants, $p < .001$. Exploratory pairwise comparisons moreover showed a vaccination rift effect among unvaccinated, $p = .002$ and recovered participants, $p < .001$. This effect was smaller but still significant among fully vaccinated participants, $p = .049$ and non-significant among partly vaccinated participants, $p = .181$.

Including message threat in the ANOVA yielded main effects of source, $F(1, 1162) = 27.55$, $p < .001$, $\eta_p^2 = .02$, and participants' vaccination status, $F(3, 1162) = 223.20$, $p < .001$, $\eta_p^2 = .37$, that were qualified by a Message Source × Participant Vaccination Status interaction, $F(3, 1162) = 3.94$, $p = .008$, $\eta_p^2 < .01$. Pairwise comparisons revealed a vaccination rift among recovered participants, p < .001 and unvaccinated participants, $p < .001$. This effect was smaller but still significant among partly vaccinated participants, $p = .032$, and non-significant among fully vaccinated participants, $p = .444$.

Including the commenter evaluation in the ANOVA yielded main effects of source, $F(1, 1162) = 5.59$, $p = .012$, $\eta_p^2 = < .01$, and participants' vaccination status, $F(3, 1162) = 192.71$, $p < .001$, $\eta_p^2 = .33$, that were qualified by a Message Source × Participant Vaccination Status interaction, $F(3, 1162) = 5.40$, $p = .001$, $\eta_p^2 = .01$. Pairwise comparisons revealed a vaccination rift among unvaccinated participants, $p < .001$, and recovered participants, $p = .014$, but not among partly vaccinated participants, $p = .479$, or fully vaccinated participants, $p = .162$.

Parallel analyses using binomial regression and including behavioral planning (i.e., requesting additional information) as the dependent measure showed a main effect of participants' vaccination status, $\chi^2(3, N = 1170) = 31.00$, $p < .001$, but no main effect of message source, $\chi^2(1, N = 1170) = 0.07$, $p = .795$, or interaction, $\chi^2(2, N = 1170) = 1.98$, $p = .577$. Ironically, those participants who were already fully vaccinated (19%) were significantly more likely to request additional information than those unvaccinated (08%), $\chi^2(1, N = 836) = 20.66$, $p < .001$, or recovered (07%), $\chi^2(1, N = 632) = 11.98$, $p = .001$. Partly vaccinated participants were also significantly more likely to request additional information than those unvaccinated (08%), $\chi^2(1, N = 538) = 8.60$, $p = .003$, or recovered (07%), $\chi^2(1, N = 334) = 6.19$, $p = .013$. No significant difference between partly and fully vaccinated participants, $\chi^2(1, N = 634) = 0.34$, $p = .562$, or recovered and unvaccinated participants emerged, $\chi^2(1, N = 536) < 0.01$, $p = .945$.

Including counterarguing as dependent measure in our ANOVA yielded a main effect of participants' vaccination status, $F(3, 1164) = 13.38$, $p < .001$, $\eta_p^2 =.04$. No main effect of source, $F(1, 1164) = 0.96$, $p = .327$, $\eta_p^2 < .01$, or Message Source × Participant Vaccination Status interaction emerged, $F(3, 1164) = 0.53$, $p = .663$, $\eta_p^2 < .01$. Fully vaccinated participants provided shorter responses than recovered, $p < .001$, or unvaccinated participants, $p < .001$. Likewise, partly vaccinated participants provided shorter responses than recovered, $p = .018$, or

unvaccinated participants, *p* = .034. Length of response neither differed between fully and partly vaccinated participants, *p* = .200, nor recovered and unvaccinated participants, *p* = .888.

Structural Equation Modelling

We next used structural equation modelling with latent variables to explore potential mechanisms regarding the rejection of calls to get vaccinated by a vaccinated source. To account for the binary outcome (i.e., requesting additional information), we used unweighted least squares (ULS) estimations[1] in our structural equation model analysis using the lavaan 0.6-9 package.[2] Comment source predicted attributed comment constructiveness (motive *B* = -.279, *SE* = .019, *p* < .001, which in turn negatively predicted comment threat, *B* = -.666, *SE* = .003, *p* < .001, and positively predicted commenter evaluations, *B* = .712, *SE* = .004, *p* < .001. Comment source also independently predicted comment threat, *B* = .058, *SE* = .015, *p* < .001, and commenter evaluations, *B* = .073, *SE* = .017, *p* < .001. Only more constructive message motive predicted higher odds that participants requested further vaccine information, *B* = .202, *SE* = .047, *p* < .001 (indirect effect: *B* = -.056, *SE* = .014, *p* < .001). The effects of message threat and commenter evaluation on requesting additional information were not significant, *p*s > .270. The model fit was excellent, CFI = .995, SRMR = .035.

We next explored the effect of participants' own vaccination status on these processes by conducting parallel SEM analyses within the four subsamples (fully vaccinated; partly vaccinated; recovered; unvaccinated). None of the mediators predicted behavioral planning among fully vaccinated participants, *B*s < .089, *SE*s = .065 - .095, *p*s > .124 (indirect effects: *B*s < .006, *SE*s = .005 - .017, *p*s > .138). Partly vaccinated participants showed opposing effects of message constructiveness, *B* = -.238, *SE* = .032, *p* < .001 (indirect effect: *B* = -.078, *SE* = .022, *p* < .001) and message threat, *B* = .104, *SE* = .030, *p* < .001 (indirect effect: *B* = .016, *SE* = .006, *p* < .005) on behavioral planning, as well as a serial mediation from message constructiveness via

message threat emerged, indirect effect: $B = .027$, $SE = .007$, $p < .001$. Recovered participants showed only an effect of message constructiveness on behavioral planning $B = 1.00$, $SE = .374$, $p = .007$ (indirect effect $B = -.240$, $SE = .093$, $p = .010$). Finally, among unvaccinated participants, only commenter evaluations influenced behavioral planning, $B = .212$, $SE = .090$, $p < .001$, albeit indirectly via message constructiveness (indirect effect: $B = -.036$, $SE = .016$, $p < .001$). The model fits were acceptable, CFIs = .963 - .995, SRMRs = .035 - .058.

Parallel analyses including counterarguing yielded similar results. In the full sample, only more constructive message motive predicted *longer* responses, $B = -.119$, $SE = .047$, $p < .001$ (indirect effect: $B = .033$, $SE = .013$, $p < .001$). The effects of message threat and commenter evaluation response length were not significant, *p*s < .107. The model fit was excellent, CFI = .993, SRMR = .049. Among fully vaccinated participants, none of the mediators predicted response length, *B*s < .035, *SE*s = .075 - .107, *p*s > .641 (indirect effects: *B*s < .011, *SE*s = .009 - .029, *p*s > .641). Among partly vaccinated participants, message threat predicted response length, $B = .127$, $SE = .055$, $p = .022$ (indirect effect: $B = .013$, $SE = .005$, $p < .001$) as well as a serial mediation from message constructiveness via message threat emerged, indirect effect: $B = .020$, $SE = .007$, $p < .001$. Among cured participants, message motive, $B = .770$, $SE = .392$, $p = .047$, and message threat, $B = .107$, $SE = .041$, $p = .009$, predicted response length. A serial mediation from message constructiveness via message threat emerged, indirect effect: $B = .035$, $SE = .014$, $p = .014$. Finally, among unvaccinated participants, none of the mediators predicted response length, *B*s < .144, *SE*s = .038 - .171, *p*s > .068 (indirect effects: *B*s < .014, *SE*s = .002 - .037, *p*s > .147). The model fits were acceptable, CFIs = .968 - .993, SRMRs = .049 - .075.

In sum, message constructiveness was the dominant overall process but participants with different vaccination status showed different effects. While the vaccination rift mediators did not influence the behavior of fully vaccinated participants, message threat emerged as the strongest

mediator of behavioral planning effects among partly vaccinated participants. Finally, unvaccinated participants' decision to engage in behavioral planning (but not counterarguing) was influenced by their evaluation of the commenter but not the length of their response. These results show that the processes underlying the vaccination rift may differ by participant group.

Appendix 2: Calls to Get Vaccinated

Unvaccinated Source

German original: *Der folgende Kommentar wurde von einer ungeimpften Person geschrieben: „Jetzt ist es wirklich fünf vor zwölf mit Corona. Die Zahlen explodieren und die Krankenhäuser laufen über. Ich habe mich selbst noch nicht impfen lassen aber will das bald nachholen. Wir ganzen Ungeimpften tragen das Virus immer noch ungebremst weiter. Wir denken nur an uns und fürchten uns eher vor einem kleinen Piecks als vor einer schlimmen Krankheit. Das muss aufhören! Wir alle sollten nicht bis zur Impfpflicht warten, sondern sofort einen Impftermin ausmachen!“*

English translation: *The following commentary was written by an unvaccinated person: „It is really urgent with Corona. The numbers are exploding and the hospitals are over full. I have not been vaccinated yet but I want to do it soon. We, all the unvaccinated, still fully pass on the virus. We only think about ourselves and are more scared of a little prickle than of a bad disease. This has to stop! We all should not wait for the vaccination mandate to take effect but make a vaccination appointment now!“*

Vaccinated Source

German original: *Der folgende Kommentar wurde von einer geimpften Person geschrieben: „Jetzt ist es wirklich fünf vor zwölf mit Corona. Die Zahlen explodieren und die Krankenhäuser laufen über. Ich habe mich selbst gleich impfen lassen und würde es jederzeit wieder tun. Aber die ganzen Ungeimpften tragen das Virus immer noch ungebremst weiter. Sie denken nur an sich und fürchten sich eher vor einem kleinen Piecks als vor einer schlimmen Krankheit. Das muss aufhören! Sie alle sollten nicht bis zur Impfpflicht warten, sondern sofort einen Impftermin ausmachen!“*

English translation: *The following commentary was written by a vaccinated person: „It is really urgent with Corona. The numbers are exploding and the hospitals are over full. I got vaccinated immediately and would do it again. But all the unvaccinated still fully pass on the virus. They only think about themselves and are more scared of a little prickle than of a bad disease. This has to stop! They all should not wait for the vaccination mandate to take effect but make a vaccination appointment now!“*

References

1. Shi D, DiStefano C, McDaniel HL, Jiang Z. Examining chi-square test statistics under conditions of large model size and ordinal data. *Structural Equation Modeling: A Multidisciplinary Journal* **25**, 924-945 (2018).

2. Rosseel Y. Lavaan: An R package for structural equation modeling and more. Version 0.5–12 (BETA). *Journal of statistical software* **48**, 1-36 (2012).

3. Thürmer JL, McCrea SM, McIntyre BM. Motivated collective defensiveness: Group members prioritize counterarguing out-group criticism over getting their work done. *Social Psychological and Personality Science* **10**, 382-392 (2019).

4. Hornsey MJ. Why being right is not enough: Predicting defensiveness in the face of group criticism. *European Review of Social Psychology* **16**, 301-334 (2005).
